# Characterization of larval gut microbiota of two endoparasitoid wasps associated with their common host, *Plutella xylostella* (Linnaeus) (Lepidoptera: Plutellidae)

**DOI:** 10.1128/spectrum.01208-24

**Published:** 2024-09-09

**Authors:** Na-na Hu, Zi-qi Wang, Si-jie Zhang, Zhi-zhi Wang, Xue-xin Chen

**Affiliations:** 1 Institute of Insect Sciences, College of Agriculture and Biotechnology, Zhejiang University, Hangzhou, China; 2 Ministry of Agriculture and Rural Affairs Key Lab of Molecular Biology of Crop Pathogens and Insect Pests, Zhejiang University, Hangzhou, China; 3 Key Laboratory of Biology of Crop Pathogens and Insects of Zhejiang Province, Zhejiang University, Hangzhou, China; 4 State Key Lab of Rice Biology and Breeding, Zhejiang University, Hangzhou, China; China Agricultural University, Beijing, China

**Keywords:** gut microbiota, *Cotesia vestalis*, *Diadromus collaris*, *Enterococcus*, microbial function

## Abstract

Insect gut microbes play important roles in digestion, metabolism, development, and environmental adaptation. Parasitoid wasps are one of the most important biological control agents in pest control, while the gut microbial species compositions and the associated functions have been poorly investigated. Two endoparasitoid wasps, *Cotesia vestalis* and *Diadromus collaris*, parasitize the larval stage and pupal stage of the diamondback moth, *Plutella xylostella*, respectively. Using whole-genome shotgun metagenomic sequencing, we characterized the gut microbial composition, diversity, and potential functional roles associated with the two parasitoid wasp larvae. The results reveal that Proteobacteria and Firmicutes are the dominant phyla in the gut of *C. vestalis* and *D. collaris* larvae, with *Rhizobium* and *Enterococcus* being the dominant genera. The putative microbial functions associated with the two parasitoid wasps might play a virtual role in assisting in consuming the host’s nutritional composition. The enriched CAZymes family genes are primarily involved in the degradation and synthesis of chitin. Despite the richness of microbial species and communities, the microbes species and the microbial community structure exhibit significant similarity between the two parasitoid wasps and between the parasitoid wasp and the host *P. xylostella*. Notably, the prevalence of the genus *Enterococcus* shared among them suggests a possible link of gut microbes between the host and their associated parasitoids. Our study offers insights into the gut microbe-based interactions between the host and parasitoid wasps for the first time, potentially paving the way for the development of an ecologically friendly biocontrol strategy against the pest *P. xylostella*.

**IMPORTANCE:**

Endoparasitoid wasps spend the majority of their lifespan within their host and heavily rely on the host’s nutrition for survival. There is limited understanding regarding the composition and physiological impacts of gut microbial communities in parasitoid wasps, particularly during the larval stage, which is directly linked to the host. Based on a thorough characterization of the gut microbe and comprehensive comparative analysis, we found the microbial species of the larval parasitoid wasp *Cotesia vestalis* and the pupal parasitoid wasp *Diadromus collaris* were similar, sharing 159 genera and 277 species, as were the microbial community structure. Certain of the dominant microbial strains of the two parasitoid wasps were similar to that of their host *Plutella xylostella* larvae, revealing host insect may affect the microbial community of the parasitoid wasps. The putative microbial functions associated with the parasitoid wasp larvae play an important role in dietary consumption.

## INTRODUCTION

Insects harbor a very diverse and complex microbiome in their guts, which includes bacteria, archaea, fungi, and protists, with bacteria being the most abundant (1–4). Gut microbial composition and activities can strongly influence insect survival and fitness by mediating the regulation of host physiology, such as development, food digestion, nutritional provision, and immunity (3, 5–7). For example, the gut bacterium, *Escherichia coli*, supports the transition of axenic larvae of *Aedes aegypti* into adult mosquitoes (8). *Bifidobacterium* and *Gilliamella* species, residing in the gut of the honey bee, are the primary degraders of hemicellulose and pectin derived from pollen (9). In some cases, like with the turtle ants (*Cephalotes* sp.) and *Dysdercus fasciatus*, gut microbes provide a broad range of benefits for insect nutrition, serving as suppliers of essential amino acids and vitamins (7, 10, 11). Insect health can be compromised by disruptions of the gut microbiota, with its diversity significantly influencing the overall health of the host (12, 13). One study demonstrated the gut microbiota of *Plutella xylostella* mediate the host’s susceptibility to Bt *Cry1Ac* protoxin by up-regulating the expression of immunity pathway genes and most antimicrobial peptide genes (14). Various factors, such as organism species and diet, can shape the composition and function of gut microbiota (15, 16). The gut microbial compositions of honey bees and bumble bees exhibit a high degree of similarity within the mega-diverse insect order Hymenoptera, whereas termites display distinct microbial community patterns (16). Diet is also one of the primary determinants of microbial community. One study has reported that the gut microbiota of wood-feeding termites were separated from those of humus and soil-feeding species (17). Thus, understanding the composition and function of gut microbes helps to manage the health and improve the survival of insects.

Endoparasitoid wasps are a large group of hymenopteran insects that lay eggs in the bodies of other arthropods that serve as hosts for the development of immature stages of wasp offspring. The parasitoid wasp adults are free living, but the larval stage of parasitoid wasp offspring is restricted inside the host body and successful development of the wasp larvae usually leads to the death of their host (18). Therefore, many parasitoid species are used as biological control agents for pest control (19). Several species of parasitoid wasps were studied for the gut microbiota of wasp adults, such as *Nasonia vitripennis* and *Cotesia vestalis,* which are involved in microbiome-mediated pesticide resistance (20, 21). Only *Nasonia* species were studied for their larval gut microbiome (22–24). Notably, holobiont interactions between shared, resident members of gut microbiome mediated hybrid lethality in the larvae of three closely related *Nasonia* species, indicating a crucial role of gut microbiota in host survival (22, 23). However, little attention has been directed toward the gut microbes of parasitoid wasp larvae and the microbe-mediated host-parasitoid wasp interaction.

The diamondback moth, *P. xylostella* (Linnaeus) (Lepidoptera: Plutellidae), is one of the most destructive pests of crucifer crops worldwide (25). *C. vestalis* (Haliday) (Hymenoptera: Braconidae) and *Diadromus collaris* (Gravenhorst) (Hymenoptera: Ichneumonidae) parasitize the larval and pupal stage of *P. xylostella*, respectively. Due to the high parasitism efficiency, the two parasitoids are widely utilized as biological control agents in suppressing the populations of *P. xylostella* (26, 27). Both parasitoids belong to Ichneumonoidea and phylogenetic analysis showed that the two parasitoids diverged approximately 124 million years ago (28). The biology and ecology of the two parasitoids have been well studied (29). The female *C. vestalis* lays its egg into the third instar larva of *P. xylostella* and the offspring feeds on the host hemolymph inside the host larva until the third instar (30). *D. collaris* prefer to parasitize the prepupa or early pupa of *P. xylostella*. The offspring of *D. collaris* developed inside the host and mainly consumed the host’s tissue such as fat bodies until the emergence of adult wasps (31, 32). As a result, these two parasitoids have distinct lifespans inside the body of *P. xylostella*. Despite this, both parasitoids tend to suppress the humoral and cellular immune defense of their host, which may have an effect on the interaction between the host and gut microbes (33). However, there is still a lack of comprehensive studies on the diversity and composition of gut microbial communities in parasitoid wasps associated with a common host, particularly for those species that have different parasitic lifestyles.

To explore the similarities and differences in the gut microbes compositions between two endoparasitoids sharing a common host, the gut microbiota of *C. vestalis* and *D. collaris* larvae was sequenced by whole-genome shotgun metagenomic sequencing. Furthermore, the 16S rRNA sequencing was also applied to analyze the potential correlation between the gut microbiome of the larval wasp and the host, *P. xylostella*.

## MATERIALS AND METHODS

### Insect rearing

The populations of *C. vestalis* (Cv) and *D. collaris* (Dc) were separately established from parasitized *P. xylostella* collected from cabbage (*Brassica* spp.) fields in Zhejiang and Yunnan Province, China. The population of *P. xylostella* was obtained from a laboratory colony and reared using an artificial diet (Keyun Biotechnology, China) as hosts. For propagation, *C. vestalis* females were induced to parasitize the third instar larvae of *P. xylostella* at a population ratio of 1:20 (wasp:host). The parasitized larvae were transported and reared on an artificial diet. Wasp cocoons of *C. vestalis* were collected and held in plastic cages until their emergence. *D. collaris* females were induced to parasitize the pupae of *P. xylostella* at a 1:10 (wasp:host) population ratio. The parasitized pupae were collected in plastic cages until their emergence. The adult wasps were fed with a 20% honey-water solution. For experiments, third instar larvae of *P. xylostella* were parasitized by exposure to a single *C. vestalis* until oviposition was observed. Similarly, pupae of *P. xylostella* were individually offered to *D. collaris* for parasitization. Insects were raised in a growth chamber (MH-352H, Sanyo, Japan) under controlled conditions of 24°C, 55% relative humidity, and 14 h light: 10 h dark photoperiod.

### Larval guts collection

**Fig 1:**
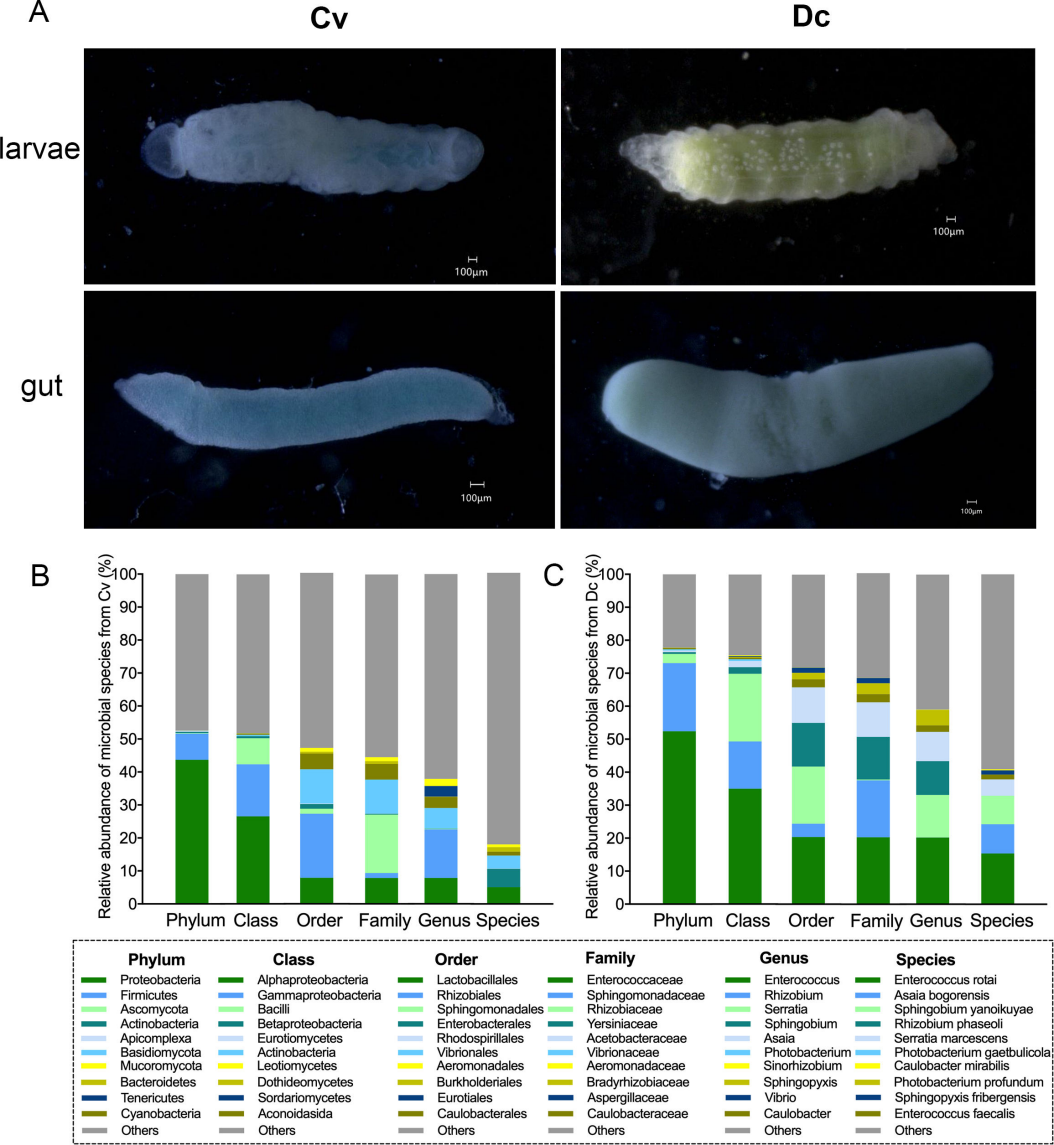
The gut and gut microbiota of parasitoid wasp larvae. (**A**) The gut of the two parasitoid wasp larvae. Scale bar: 100 µm. (**B and C**) Gut microbiota composition of the two parasitoid wasps. Overview of the top 10 most abundant microbes in the larval gut of *C. vestalis* (**B**) and *D. collaris* (**C**) at each taxonomic rank (i.e., phylum, class, order, family, genus, and species).

After surface sterilizing with 75% ethanol for 90 s, the second instar *C. vestalis* larva (the last stage inside the host) was collected from late-stage fourth instar (4L) parasitized *P. xylostella* larvae and then rinsed twice with sterilized PBS buffer (NaCl 136.89 mM; KCl 2.67 mM; Na_2_HPO_4_ 8.1 mM; KH_2_PO_4_ 1.76 mM; pH 7.4). Similarly, *P. xylostella* pupae were dissected 5 days after parasitization by *D. collaris*, and *D. collaris* larvae at the last stage (fourth instar) were collected. Being transported to a sterile Petri dish, the parasitoid wasp larva was then dissected separately and the guts of *C. vestalis* or *D. collaris* were collected (Fig. 1A) and stored in sterile PBS-EDTA solution (PBS buffer containing 5‰ EDTA) at −80°C before DNA extraction. Each experiment treatment was repeated three times, with 50–60 guts in each replicate.

To collect the midgut contents of parasitized *P. xylostella*, third instar larvae of *P. xylostella* were exposed to *C. vestalis* until parasitization was observed. Both parasitized (P) and non-parasitized (N) middle fourth (4M) instar and 4L instar larvae of *P. xylostella* were collected. The larvae were surface-sterilized with 75% ethanol for 90 s, rinsed twice with sterilized PBS buffer, and dissected in cold sterile PBS-EDTA solution under aseptic conditions. The midguts were removed, rinsed, and stored in the sterile PBS-EDTA solution at −80°C before DNA extraction. Each sample group contains 50 midguts of *P. xylostella*.

### DNA extraction and sequencing

For metagenomic sequencing, the total DNA of larval *C. vestalis* or *D. collaris* guts was extracted using the EZNA Stool DNA Kit (D4015, Omega, Inc., USA) according to the manufacturer’s instructions. The quality and quantity of extracted DNA were determined by spectrophotometer analysis (NanoDrop ND-1000, NanoDrop Technologies, Willmington, DE) and agarose gel electrophoresis (1.2%), respectively. Then samples were subjected to Illumina sequencing (NovaSeq, Shanghai, China) for high throughput sequencing.

For 16S rRNA sequencing, gut samples of parasitized and non-parasitized *P. xylostella* larvae (4M and 4L), and *C. vestalis* (2L) were sequenced using MiSeq technology. Briefly, the V4-V5 region of the bacterial 16S rRNA gene was amplified with universal primers 515F (5′-GTGCCAGCMGCCGCGGTAA-3′) and 926R (5′-CCGTCAATTCMTTTGAGTTT-3′). The resulting amplicons were then sent to TinyGene Bio-Tech (Shanghai) Co., Ltd. and subjected to 16S rDNA sequencing using the Illumina MiSeq platform.

### Metagenomics data assembly

To obtain a valid read the adapter sequences were first removed from reads with cutadapt (v1.2.1) (34). Secondly, low-quality reads were trimmed by fastp (0.20.0), and accessed using a sliding window algorithm. Thirdly, to remove the host sequence, the reads were mapped to the reference insect genome (28) using Burrows-Wheeler Alignment tool (35). After quality filtering, clean reads of the metagenomic data set were *de novo* assembled into contigs using the megahit Next generation sequencing*de novo* assembler (36). Then, contigs were generated after constructing a De Bruijn graph and then joined into scaffolds with IDBA (37). After quality evaluation, the Scaffolds/Scaftigs (≥200 bp) were selected for the MetaGeneMark analysis to predict an open reading frame (ORF) (38). Then, CD-HIT was used to remove redundancy, and the reads at 90% of identity were used to cluster (39). The longest sequence becomes the representative of the first cluster and was used to generate non-redundant protein data sets. Then, read counts were produced using htseq-count (40).

### Microbial taxonomic profiling

For the analysis of metagenomic sequencing data, the microbial taxonomy was annotated by assigning reads to nodes in the NCBI taxonomy using MEGAN (41). Combined with NCBI’s species classification system, species annotation information at different taxonomic levels was obtained. The relative abundance of phylum, class, order, family, genus, and species level between groups was calculated by Quantitative Insights into Microbial Ecology (QIIME) (42) and visualized as cumulative histograms by GraphPad Prism 6.

For the analysis of 16S rRNA sequencing data, FASTA files were filtered to a minimum read length of 200 bp and were clustered into operational taxonomic units (OTUs) at 97% sequence identity using Mothur software (http://www.mothur.org). OTU taxonomies were determined based on the SILVA database (v132, http://www.arb-silva.de).

### Microbial community diversity and structure analyses

The microbial species richness (i.e., ACE, Chao1) and diversity (i.e., Shannon and Simpson) indices of microbial community were estimated as the indicators for alpha diversity using QIIME. The construction of the histogram was applied to compare the alpha diversity values between the two groups, and the statistical analysis was performed using the two-tailed unpaired *t*-test. The similarity of microbial community structure among groups was examined by beta diversity analysis. The permutation-based permutational multivariate analysis of variance (PERMANOVA) was performed by R software, and 999 displacement tests were performed to determine whether the differences were statistically significant (43). Overlapping and unique microbial genera were generated by the Venn Diagram package in R software (44). A heatmap of the abundant gut microbial taxa between the two groups was plotted using R software, the hierarchical clustering analysis of all samples to exhibit the similarity among samples, which was performed with unweighted pair-group method with arithmetic means (UPGMA) and the Bray-Curtis similarity. An extended error bar in Statistical Analysis of Metagenomic Profiles (STAMP) software was utilized to identify taxonomic changes of dominant microbes with significant differences between the two groups (45). The significant difference between the two groups was analyzed by Welch’s *t*-test with Benjamini-Hochberg FDR correction *P* < 0.05.

### Microbial functional profiling

The predicted peptide sequences were annotated using the Kyoto Encyclopedia of Genes and Genomes (KEGG) database by DIAMOND and DataBase for automated Carbohydrate-active enzyme Annotation with the optimized e-value threshold of 1E-3, respectively (46–48). The relative abundance of each KEGG orthologous group (KO) and carbohydrate-active enzymes (CAZymes) were calculated from the abundance of its genes and visualized as cumulative histograms by GraphPad Prism 6. An extended error bar in STAMP was utilized to identify functional changes in the enriched pathways or CAZymes with significant differences between the two groups. A heatmap of the top 20 predicted functions by CAZymes families of the two groups was plotted using R software, and the UPGMA trees were based on Bray-Curtis distances. Linear discriminant analysis effect size (LefSe) analysis was performed to identify specific markers of KEGG pathways between groups using the online LefSe workflow on the Hutlab Galaxy platform (http://huttenhower.sph.harvard.edu/galaxy/). We also explored the correlation between gut microbiota and CAZymes or KEGG pathways by Spearman correlation analysis (49–52). The correlation analysis was assessed and visualized by the Genescloud tools, a free online platform for data analysis (https://www.genescloud.cn).

### Quantification of bacterial communities

The total DNA of each replicate was extracted with QIAamp DNA Mini Kit (Qiagen, Hilden, Germany) from pools of five midguts of the parasitized and non-parasitized (4M and 4L) larvae of *P. xylostella*. The DNA extraction was performed following the modification recommended by the manufacturer (53). To quantify bacterial load, quantitative PCR (qPCR) was performed on DNA extracted. The 16S rRNA was amplified using a pair of universal primers (F: 5′-TCCTACGGGAGGCAGCAGT-3′, R: 5′-GGACTACCAGGGTATCTAATCCTGTT-3′) (54). A pair of *Enterococcus-*specific primers (F: 5′-CCATCAGAGGGGGATAACACTT-3′, R: 5′-TCAGTGACACCCGAAAGCG-3′) were designed for this study targeting an approximately 178 bp fragment of the 16S rRNA-encoding gene of *Enterococcus* sp. qPCR reactions were performed in a 20 µL reaction mix containing 10 µL 2×SYBR qPCR Mix (TOYOBO), 0.6 µL each of 10 mM primers, 0.2 µL of template DNA, and 8.6 µL H_2_O. The cycling conditions used were 95°C for 1 min followed by 40 cycles of 95°C for 15 s and 60°C for 30 s. The load of midgut bacteria was determined by a qPCR assay. qPCR was conducted with three independent replicates. The *P. xylostella β-actin* (GenBank acc. no.: JN410820) was used as an internal control. The relative expression level was calculated using the 2^−△△Ct^ method (14, 54).

## RESULTS

### Gut microbial community composition of the two parasitoid wasp larvae

The metagenomic sequencing of *C. vestalis* and *D. collaris* larval guts separately generated 26.26 and 14.39 Gb raw data. *De novo* assembly annotated 29,007 and 130,310 ORFs with an average length of 409.06 bp and 276.67 bp from *C. vestalis* and *D. collaris*, respectively (Table S1). In total, we identified 22 microbial phyla, 39 classes, 81 orders, 152 families, 330 genera, and 720 species in the guts of *C. vestalis* larvae (Table S2). At the phylum level, the dominant phylum is Proteobacteria accounting for 43.68% of the total phyla, followed by Firmicutes (7.96%), and the relative abundance of the remaining phyla is less than 1%. Within the Proteobacteria, the most abundant class is Alpha-proteobacteria, with an average of 26.54%, followed by Gamma-proteobacteria (15.82%) and Beta-proteobacteria (0.61%). The phylum Firmicutes primarily consists of Bacilli, accounting for 7.92% of the total microbial compositions. At the genus level, the most prevalent genera are *Rhizobium* (14.78%), *Enterococcus* (7.85%), *Photobacterium* (6.23%), *Caulobacter* (3.37%), *Vibrio* (3.20%), *Sinorhizobium* (2.16%), *Aeromonas* (1.14%), and *Sphingomonas* (1.11%). The dominant species include *Rhizobium phaseoli* (5.58%), *Enterococcus rotai* (5.01%), *Photobacterium gaetbulicola* (3.94%), *Photobacterium profundum* (1.37%), *Enterococcus faecalis* (1.16%), *Rhizobium leguminosarum* (1.03%), and *Aeromonas veronii* (0.93%) (Fig. 1B).

A total of 590 microbial species were obtained in the gut microbiota of *D. collaris* larvae. These species were subdivided into 23 phyla, 49 classes, 100 orders, 163 families, and 298 genera (Table S2). At the phylum level, the top five phyla include Proteobacteria, Firmicutes, Ascomycota, Actinobacteria, and Apicomplexa. Proteobacteria and Firmicutes, as the most prominent phyla, account for 73.08% of the total phyla. The Sphingomonadales (17.32%), Enterobacterales (13.27%), Rhodospirillales (10.78%), Rhizobiales (4.07%), Caulobacterales (2.44%), and Burkholderiales (1.98%) from Proteobacteria, Lactobacillales (20.38%) from Firmicutes, and Eurotiales (1.59%) from Ascomycota dominate the microbes at the order level. At the genus level, the gut microbiota was dominated by *Enterococcus* (20.24%), followed by *Serratia* (12.87%), *Sphingobium* (10.24%), *Asaia* (8.88%), *Sphingopyxis* (4.68%), *Caulobacter* (1.94%), *Aspergillus* (1.52%), *Bradyrhizobium* (1.41%), and *Acidovorax* (1.03%), whereas the other genera each comprises less than 1%. The dominant microbial species include *E. rotai* (15.39%), *Asaia bogorensis* (8.88%), *Sphingobium yanoikuyae* (8.63%), *Serratia marcescens* (4.96%), *E. faecalis* (1.45%) and *Sphingopyxis fribergensis* (1.29%) (Fig. 1C).

### Similarity of gut microbial community between the two parasitoid wasp larvae

**Fig 2:**
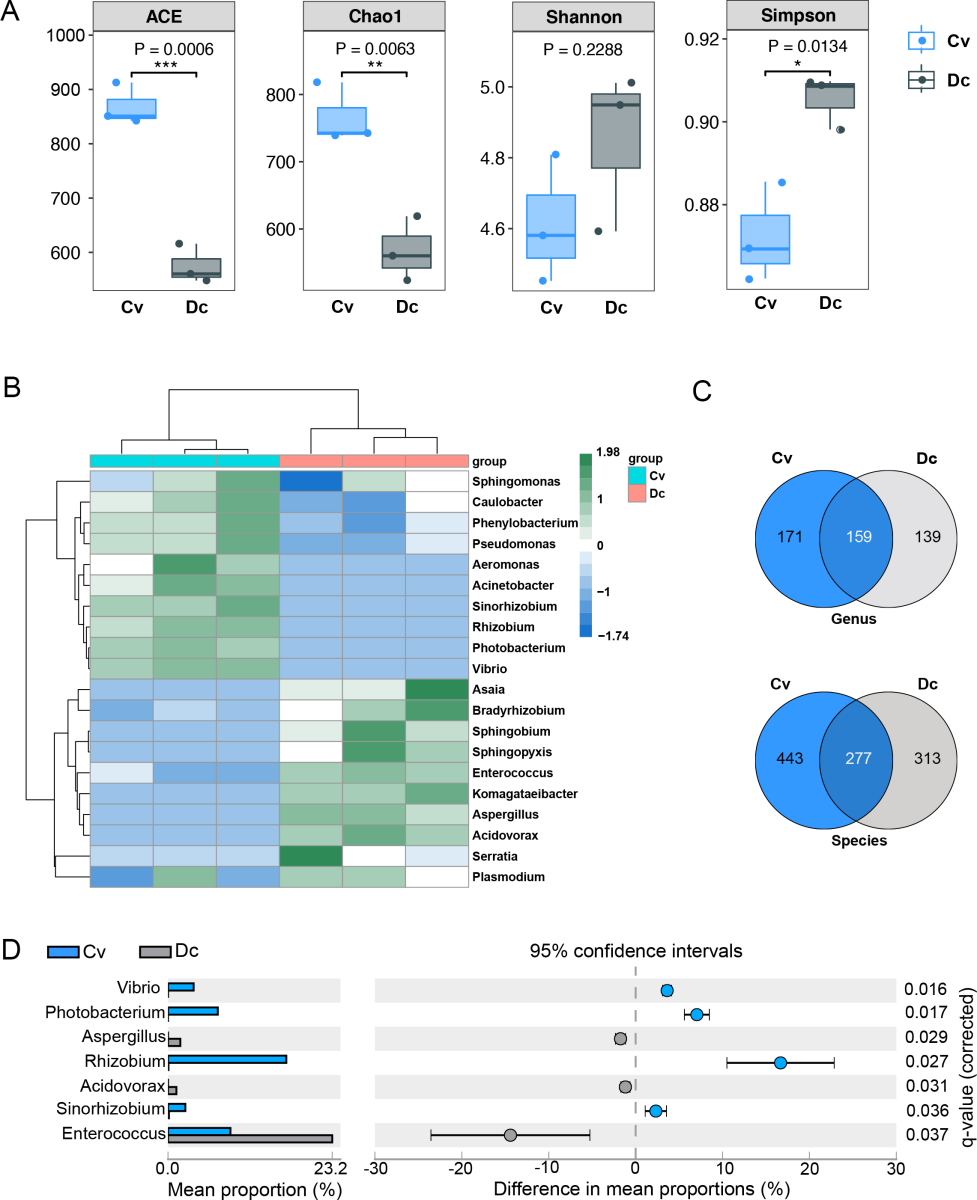
Comparison of the gut microbial community between larval Cv and Dc. (**A**) Statistics of alpha diversity indices of gut microbiota between larval Cv and Dc. Chao1 and ACE indexes were used to determine community richness, and the Shannon and Simpson indexes were used to determine community diversity. Data were analyzed using the two-tailed unpaired *t*-test (*, *P* < 0.05; **, *P* < 0.01; ***, *P* < 0.001). (**B**) Heatmap of top 20 abundant genera in the gut microbial communities of larval Cv and Dc. UPGMA trees based on Bray-Curtis distances. The heatmap scale displays the row Z score calculated based on microbial community distributions. The Z score measures how many standard deviations a data point is from the mean in a distribution. (**C**) Venn analysis of the gut microbes of larval Cv and Dc at the genus and species level. (**D**) An extended error bar plot showing the significant abundance of dominant microbial genera of larval Cv and Dc. A significant difference (*P* < 0.05) between the two groups was analyzed by Welch’s *t*-test with Benjamini-Hochberg FDR correction.

To compare the gut microbial composition between *C. vestalis* and *D. collaris* larvae, the alpha diversity and beta diversity of the microbial community were analyzed. The results showed the richness of microbial species in *C. vestalis* is significantly higher than that in *D. collaris* as indicated by the ACE (*P* = 0.0006) and Chao1 (*P* = 0.0063) values (Fig. 2A; Table S3). However, the Simpson diversity index is significantly higher in *D. collaris* (*P* = 0.0134), suggesting the gut microbiota of this parasitoid wasp possess more diverse communities with greater richness and abundance. Upon comparing the Shannon diversity index between the two groups, no significant difference is discovered in the richness and evenness of microbial diversity (*P* = 0.2288) (Fig. 2A; Table S3). Furthermore, our results demonstrated no significant difference in the beta diversity of microbial communities between the two parasitoid wasps at different classification levels (PERMANOVA, *P* = 0.1) (Table S4).

To visualize the dynamic patterns of the dominant gut microbes in the parasitoid wasps, the top 20 most abundant taxa were clustered and plotted in a heatmap at the genus level. The dendrogram showed *C. vestalis* and *D. collaris* are grouped into different branches by hierarchical clustering analysis, suggesting the dynamic patterns of the dominant gut microbes were different (Fig. 2B). However, there are 159 genera and 277 species of microbes shared between the two parasitoids (Fig. 2C; Table S5). The microbial genera *Enterococcus* and *Rhizobium* predominate within the microbial communities of the two parasitoid wasps, followed by the genera *Serratia*, *Sphingobium,* and *Caulobacter*. Unique genera in *C. vestalis* and *D. collaris* account for 36.46% (171/469 genera) and 29.64% (139/469 genera) of the total number of microbial genera, respectively. *Photobacterium* and *Asaia* are identified as the dominant unique microbial genera in *C. vestalis* and *D. collaris*, respectively (Table S6). Compared with *D. collaris*, there is a significantly higher level of *Rhizobium*, *Photobacterium*, *Vibrio,* and *Sinorhizobium* in *C. vestalis*. On the contrary, the relative abundances of *Enterococcus* are significantly higher in *D. collaris* (Fig. 2D). Our results show abundance differences in the dominant gut microbial genera of the two parasitoid larvae.

### Potential functions of gut microbiota according to the KEGG analysis

**Fig 3:**
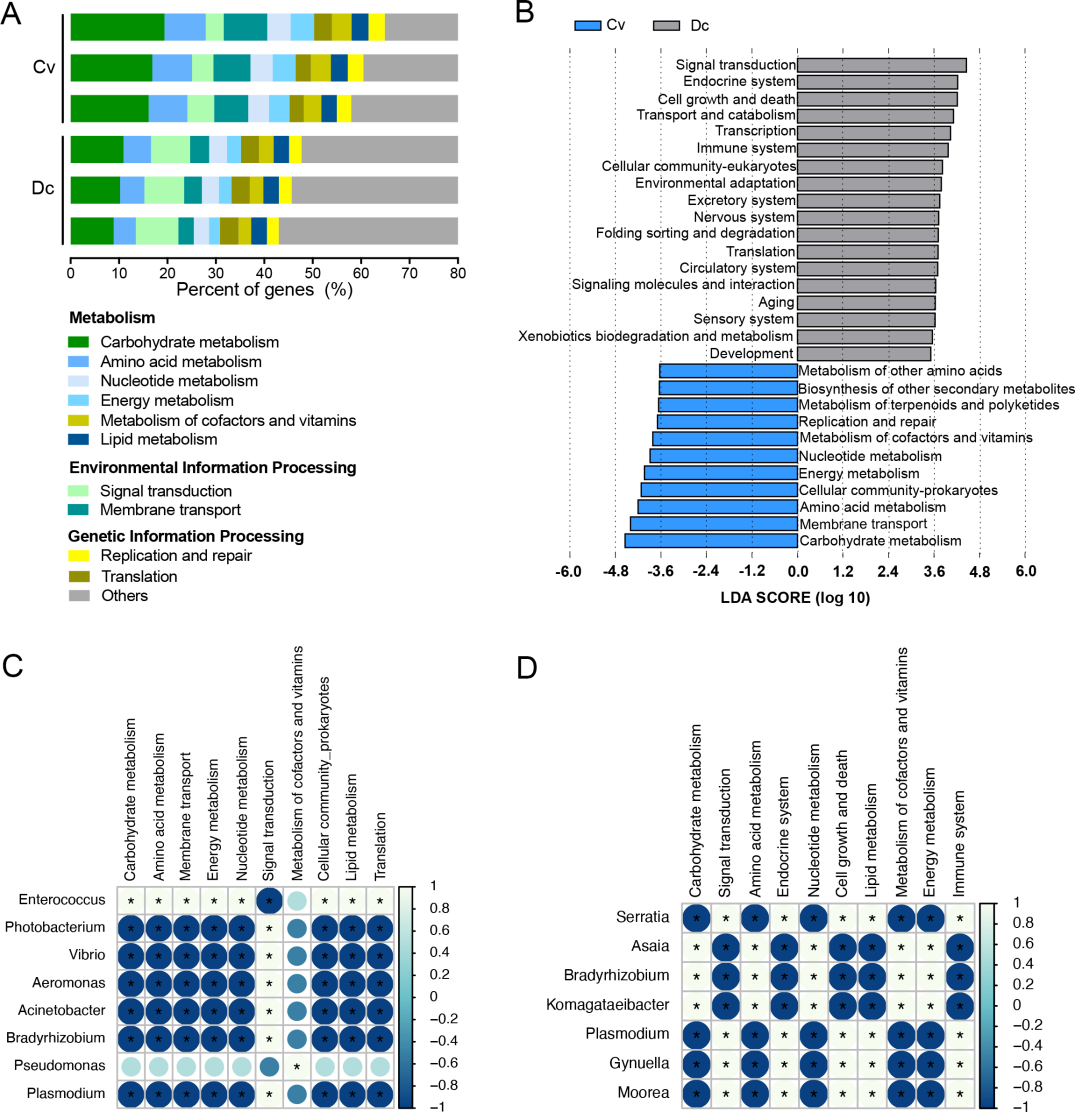
Overview of functional profiles for the gut microbes based on KEGG pathways and their correlations with selected microbes. (**A**) Relative abundance of the top 10 abundant KEGG pathways at level 2 in the gut metagenome of larval Cv and Dc. (**B**) Twenty-nine significantly different pathways with linear discriminant analysis (LDA) threshold > 2, *P* < 0.05. The LDA scores were estimated between Cv and Dc in the LEfSe analysis. (**C**) Relationship between functional profile and selected microbes within the *C. vestalis* group. (**D**) Relationship between functional profile and selected microbes within the *D. collaris* group. Correlation analyses were conducted using Spearman’s rank correction. The heatmap scale ranges from −1 (deep blue, a perfect negative correlation) to 1 (light blue, a perfect positive correlation). “*” indicates significant correlations |R| > 0.5 and *P* < 0.05.

The potential biological functions of gut microbiota were analyzed using the KEGG database. In *C. vestalis*, more than 50% of the genes are enriched to metabolism, followed by environmental information processing (12.54%) and genetic information processing (8.57%). The most enriched metabolism pathways are carbohydrate metabolism, accounting for 17.46%, followed by amino acid metabolism (8.23%), membrane transport (7.90%), energy metabolism (4.61%), and nucleotide metabolism (4.57%). For *D. collaris*, nearly 33% of the genes are mapped onto metabolism, followed by genes related to human diseases (17.40%) and organismal systems (12.49%). The primary enrichment pathways are involved in carbohydrate metabolism (10.02%), signal transduction (8.35%), amino acid metabolism (5.10%), and endocrine system (4.19%) (Fig. 3A). Significantly different pathways between the gut metagenome of *C. vestalis* and *D. collaris* were observed by LEfSe analysis (Fig. 3B). Genes in *C. vestalis* gut microbes are enriched to pathways associated with the metabolism of carbohydrates, amino acids, cofactors and vitamins, while *D. collaris* has more abundant KOs related to the system of digestive, immune, endocrine, nervous, and cell growth and death.

Correlation analyses between active microbial taxa and KEGG pathways revealed that *Enterococcus* in *C. vestalis* is positively and significantly correlated with most of the differential KEGG pathways, such as metabolism of carbohydrates, amino acid, energy, etc. (except signal transduction). *Photobacterium* is positively and significantly correlated with signal transduction, while negatively correlated with other pathways (Fig. 3C). Besides, the most predominant genus *Rhizobium* is positively and significantly correlated with xenobiotics biodegradation and metabolism, cell growth and death, infectious diseases, etc. (Table S7). In *D. collaris*, *Serratia* shows significantly positive correlations with cell growth and death, lipid metabolism, endocrine system, immune system, etc., while it negatively correlates with carbohydrate metabolism, amino acid metabolism, nucleotide metabolism, metabolism of cofactors, and vitamins (Fig. 3D). Compared with that in *C. vestalis*, *Enterococcus*-associated functions in *D. collaris* are mainly associated with pathways such as cancers: specific types, replication and repair, and aging (Table S8), suggesting that close related microbial species may play distinctive roles in different hosts.

### Potential functions of gut microbiota according to the CAZymes database

**Fig 4:**
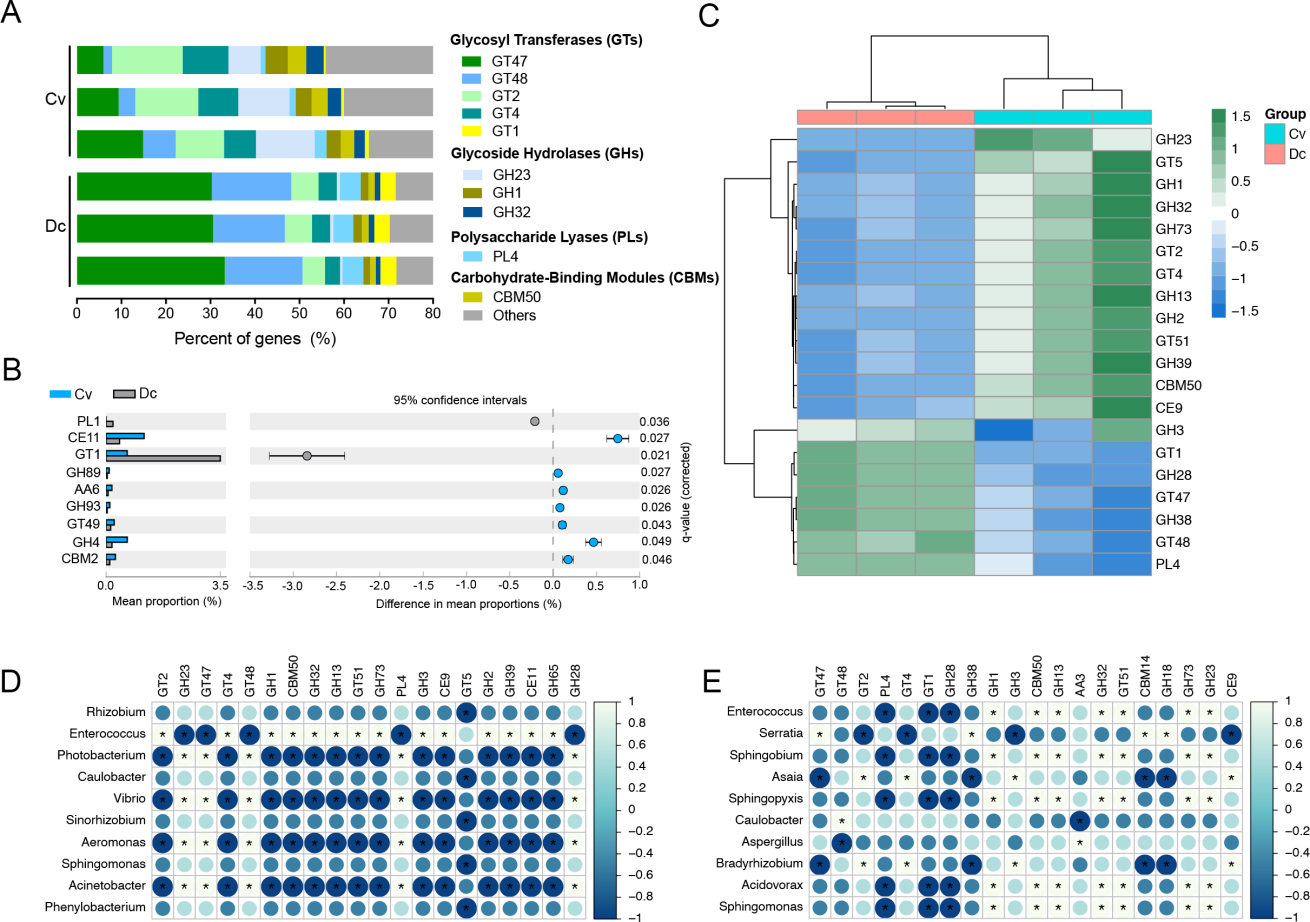
Overview of functional profiles for the gut microbes based on CAZymes families at level 2 and their correlations with selected microbes. (A) Relative abundance of top 10 abundant classes of microbial CAZymes at level 2 in the gut metagenome of larval Cv and Dc*.* (B) An extended error bar plot showing the significant abundance of various classes of microbial CAZymes at level 2 in the gut metagenome of Cv and Dc. A significant difference (*P* < 0.05) between the two groups was analyzed by Welch’s *t*-test with Benjamini-Hochberg FDR correction. (C) Heatmap of the top 20 predicted functions by CAZymes families in the gut metagenome of larval Cv and Dc*.* UPGMA trees based on Bray-Curtis distances. The heatmap scale displays the row Z score calculated based on microbial functional distributions. The Z score measures how many standard deviations a data point is from the mean in a distribution. (D) Relationship between CAZymes families and selected microbes within the *C. vestalis* group. (E) Relationship between CAZymes families and selected microbes within the *D. collaris* group. Correlation analyses were conducted using Spearman’s rank correction. The heatmap scale ranges from −1 (deep blue, a perfect negative correlation) to 1 (light blue, a perfect positive correlation). “*” indicates significant correlations |R| > 0.5 and *P* < 0.05.

The genes mapped to the CAZymes database were extracted to investigate the gut microbiota contributing to the carbohydrate-active enzymes. There are six classes of CAZymes, mainly glycosyl transferases (GTs), glycoside hydrolases (GHs), carbohydrate-binding modules (CBMs), carbohydrate esterase (CEs), polysaccharide lyases (PLs), and auxiliary activities (AAs) families (55). In *C. vestalis*, candidate sequences that belong to GTs (45.61%) and GHs (40.22%) families are the most abundant, followed by CBMs (7.10%) and CEs (4.39%) families. The most frequently occurring GT families in the gut metagenome of *C. vestalis* are GT2 (13.62%), GT47 (10.08%), and GT4 (8.81%). The most predominant GH families are GH23 (10.63%), followed by GH1 (3.99%) and GH32 (3.12%). In *D. collaris*, the enzymes belonging to GTs are predominant, representing approximately 66.37% of all known families, with GT47 (31.38%) and GT48 (17.14%) displaying the highest abundance. Additionally, GHs (19.98%) constitute the second largest family, with GH28 (3.02%) and GH38 (2.25%) being the dominant enzymes (Fig. 4A). The CAZymes from CE11 and GH4 are more abundant in *C. vestalis*, while GT1 is significantly higher in *D. collaris* (Fig. 4B). The divided clusters in the dendrogram suggest different dynamic patterns of the dominant enzymes between *C. vestalis* and *D. collaris* (Fig. 4C).

To identify which microbial genera play an essential role in the carbohydrate-active enzyme families, spearman’s rank correlations were analyzed between the top 10 genera and the CAZymes families. *Enterococcus* in *C. vestalis* is positively and significantly correlated with most of the differential CAZymes families, with the main production of GT2, GT4, and GH1. The results show that GH23, GT47, and GT48 are significantly positive correlations with *Photobacterium*, *Vibrio*, *Aeromonas,* and *Acinetobacter* (Fig. 4D). In *D. collaris*, *Enterococcus*, *Sphingobium,* and *Sphingopyxis* are positively and significantly correlated with CAZymes families GH1, CBM50, GH13, and GH32, while negatively correlated with PL4, GT1, and GH28 (Fig. 4E). As the second dominant genus, *Serratia* exhibits a positive and significant correlation with CAZymes families GT47, while displaying a negative correlation with GT2 (Fig. 4E).

### Comparison of gut microbiota between *C. vestalis* larvae and its host *P. xylostella*

**Fig 5:**
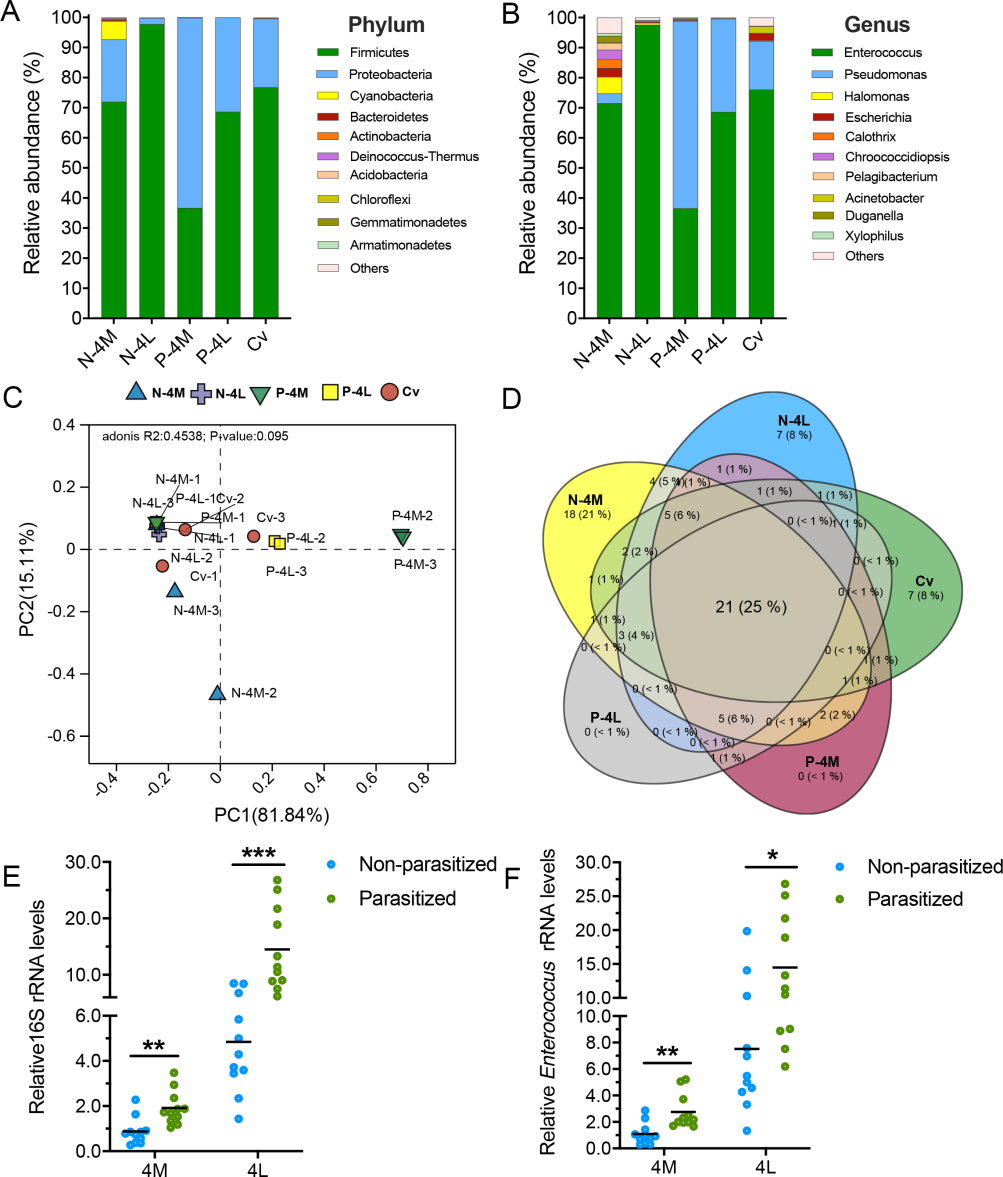
Comparative assessment of the gut microbiota among non-parasitized *P. xylostella*, parasitized *P. xylostella,* and *C. vestalis*. (**A and B**) Phylusm-level (**A**) and genus-level (**B**) comparison of relative abundances of the microbial communities of *P. xylostella* and associated *C. vestalis* larvae. (**C**) PCoA plot based on microbial community structure (Bray-Curtis dissimilarity distance). (**D**) Venn diagram of microbes showing the common and unique genera among groups. N-4M, non-parasitized middle fourth instar larvae of *P. xylostella*; P-4M, parasitized middle fourth instar larvae of *P. xylostella*; N-4L, non-parasitized late fourth instar larvae of *P. xylostella*; P-4L, parasitized late fourth instar larvae of *P. xylostella*; Cv, larval *C. vestalis*. A load of total bacteria (**E**) and *Enterococcus* sp. (**F**) in the midgut of non-parasitized and parasitized *P. xylostella* larvae. Quantification was by 16S rRNA gene-based qPCR analysis and normalized against *P. xylostella β-actin*. 4M, middle fourth instar larvae; 4L, late fourth instar larvae; The horizontal line represents the mean value of the results. One dot represents five guts. Each treatment contains eleven biological replicates. Significance was analyzed by the two-tailed unpaired *t*-test (*, *P* < 0.05; **, *P* < 0.01; ***, *P* < 0.001).

To analyze the potential correlation between the gut microbiome of the larval wasp and its host larvae, the OTUs were obtained from the parasitized *P. xylostella* larvae, the non-parasitized *P. xylostella* larvae, and the parasitoid *C. vestalis* larvae by 16S rRNA sequencing. At the phylum level, Firmicutes and Proteobacteria dominate microbial communities of both *C. vestalis* and its host *P. xylostella* (Fig. 5A). In both parasitized and non-parasitized *P. xylostella*, *Enterococcus* dominates across different developmental stages, accounting for 71.60% and 97.56% at 4M and 4L, respectively. It is also the prevalent genus in the gut of *C. vestalis* larvae (76.11%), suggesting a high abundance of *Enterococcus* in the gut microbiome of both *C. vestalis* and the host *P. xylostella*. Besides, a high proportion of *Pseudomonas* is present in the midgut of parasitized *P. xylostella* and *C. vestalis* (Fig. 5B). We also compared the microbial community composition between the parasitoid wasp *C. vestalis* and their host *P. xylostella*. PCoA based on Bray-Curtis distance shows no significant difference among *C. vestalis*, non-parasitized and parasitized *P. xylostella*, indicating high similarities between the wasp larvae and parasitized *P. xylostella* microbiota (PERMANOVA, R^2^ = 0.4538, *P* = 0.095) (Fig. 5C). The Venn diagrams results show that 21 genera are shared between the two species, accounting for 25% of the total number of genera (Fig. 5D). qPCR results also demonstrate that the total bacteria load and relative abundance of *Enterococcus* are significantly increased in the midgut of parasitized *P. xylostella* larvae compared to that from the non-parasitized *P. xylostella* (Fig. 5E and F). These results indicate that parasitoid wasp larvae may share and/or obtain host microbes during their development within parasitized host larvae.

## DISCUSSION

The insect gut microbiota plays a significant role in digestion, development, and pathogen resistance (56). In this study, the larvae of *C. vestalis* and *D. collaris* were applied to investigate the microbial similarity, variation, and diversity of parasitoid wasps associated with a common host. Functional analyses reveal potential effects of gut microbial communities, especially the dominant species composition in each parasitoid wasp. Additionally, the similarity of gut microbes between the host larvae and the parasitoid wasp *C. vestalis* larvae suggests a possible link of gut microbes between the host and their associated parasitoids.

High throughput sequencing technologies have been used to characterize the whole-body general microbial community present in parasitoid wasps such as the *Eretmocerus* species *Eretmocerus mundus* and *Eretmocerus eremicus*, *Encarsia Formosa* and *Telenomus tridentatus* (57, 58). The predominant bacteria found in the majority of parasitoid wasps belong to the Proteobacteria and Firmicutes phyla (59). Our results confirmed Proteobacteria and Firmicutes were the predominant members of gut microbes, accounting for approximately >52% of the total microbial compositions of larval parasitoids, consistent with previous reports in the whole-body of parasitoid adults (57, 59–62).

The characterization of the larval gut microbiota of *C. vestalis* and *D. collaris* revealed the representative genera *Enterococcus*, which is a prevalent family in the gut of numerous insects, may play a vital role in the parasitoid wasps. Recent studies have shown that *Enterococcus* plays an essential role in the body weight or survival of *Samia ricini* and *Spodoptera frugiperda* on the pinto bean diet (63, 64). Our results showed *Enterococcus* might participate in carbohydrate and amino acid metabolism in parasitoids, suggesting that it may act as a probiotic in parasitoid wasps, contributing to the wasp’s health and growth. At the species level, *E. rotai* was the dominant microbe in the two parasitoids. Though it has been found mainly in invertebrates like mosquitoes, its potential function is largely unknown (65, 66). The closely related species are involved in digestion and nutrient supply. For example, *Enterococcus mundtii* isolated from the midgut of *P. xylostella* could effectively utilize a wide range of carbon compounds (67); *Enterococcus casseliflavus* from *Bombyx mori* gut could improve the production of an essential amino acid L-tryptophan, which was required in several physiological processes of insects (68). However, the potential function of *Enterococcus*, especially *E. rotai*, in parasitoid wasp larvae is currently ambiguous and requires further investigation.

A predominance of members belonging to the genus *Rhizobium* in *C. vestalis* with *R. phaseoli* being the most abundant species. *R. phaseoli* was well known for its role in plant growth promotion (69), while there was no information available on its presence and function in insects. Previous studies showed the gut bacteria *Rhizobium* of *Trichoplusia ni* shows a potential ability to degrade organic alkaloids (70, 71). Besides, *Rhizobium* detected as a nitrogen-recycling endosymbiont in the gut of *Tetraponera* ants, has convergently evolved enzymatic pathways to transform nitrogenous metabolites into amino acid precursors (72). Our analyses supported *Rhizobium* from the gut microbiota of *C. vestalis* plays an essential role in xenobiotics biodegradation and metabolism, which would help to counter the toxic or harmful phytochemicals from the diet.

The larva gut of *D. collaris* commonly carries *Serratia*, mainly *S. marcescens*, which frequently acts as an opportunistic pathogen in insects (73). In our study, *Serratia* had significantly positive correlations with the immune system in the *D. collaris*. A similar research found *S. marcescens* Y1 from *Anopheles sinensis* gut may render the mosquito resistant to *Plasmodium berghei* infection through the activation of the host immune system (74). A previous study also showed *Serratia* in pea aphids may act as an assistant since it facilitated the growth and development of aphids by enhancing fatty acid biosynthesis (75). As developing inside *P. xylostella* pupae, lipid is the main nutrition source of *D. collaris* larvae. The involvement of *Serratia* in lipid metabolism may contribute to adapting to the lipid-richness pupae of *P. xylostella*. However, the potential functions of *S. marcescens* in the parasitoid, whether it is a mutualist or pathogen, still need further research.

The gut microbiota plays virtual roles in diet digestion, such as lipids, protein, and sugars of the insect host of *Anticarsia gemmatalis* and honey bees (76, 77). The larvae of *C. vestalis* fed primarily on the host hemolymph, a nutrient-rich medium containing carbohydrates such as trehalose and glucose, proteins, and various amino acids (78). As a result, putative functions associated with the metabolism of carbohydrates, amino acids, energy, nucleotides, and vitamins were enriched in the gut microbiota of *C. vestalis*. In contrast, *D. collaris* completed its development inside the pupa stage of the host and consumed the caterpillar before pupation (32). The putative microbial functions such as the digestive system, endocrine system, xenobiotic biodegradation and metabolism, cell growth and death were more active in *D. collaris*. Based on the putative microbial functions associated with the parasitoids, we speculated the differences in the nutritional composition variances between the larval and pupal hosts may contribute to the microbial difference between the two parasitoid wasps. It also noted that more abundant bacterial species and quantity were present in the larval host than in pupal ones (79). The variation in microbes at different stages of *P. xylostella* may account for the distinction in gut microbes between the two parasitoids. Accordingly, we could not exclude the possibility that parasitic ecological niches may influence the composition of gut microbes of these two parasitoid wasps.

Chitin is an important constituent of midgut cells and peritrophic matrices, which are crucial for insect growth and development (80). The presence of gut microbiota is required for the synthesis of structurally integrated peritrophic matrices of insects (81). The expression of peritrophins and chitin synthetase in the midgut was significantly reduced following antibiotic treatment (82). Our study showed the gut microbiota of parasitoid wasps contributing to most CAZymes families genes mainly take part in the degradation and synthesis of chitin. For example, the most predominant CAZyme family of *C. vestalis*, GT2, was homologous to chitin synthases in bacteria. Besides, GH23 found to be abundant in *C. vestalis*, was identified as chitinase in *Ralstonia* sp. A-471 (83, 84). The most abundant CAZyme genes identified in the gut metagenome of *D. collaris* were related to GT47. The N-acetylglucosaminyl transferase (GT47) such as the NodC protein was reported to share sequence homology with chitin synthase (85). Moreover, insect feeding is the source of complex and diverse foreign microorganisms including pathogenic microorganisms. The gut commensal microbiota of insects is also involved in the immune defense response, which is crucial for resisting and eliminating foreign pathogens (86). The CAZymses from GT48 display high abundance in *D. collaris*, which was known activities in putative (1, 3)-β-glucan synthase, an enzyme that synthesizes β-glucan (87). Pattern recognition proteins binding to β-glucan have been implicated in the activation of innate defense reactions, which would help to destroy or immobilize invading pathogens from the putrefaction of unconsumed host tissue during the feeding period of larvae.

Due to the close relationship with their host, parasitoids may exhibit substantial communication with their DBM hosts including symbiotic microbes. The composition of symbiotic bacteria in the whole body of *Lysiphlebia japonica* larvae was highly similar to that of their aphid host *Aphis gosypii*, especially the high abundance of *Buchnera* (88). Host species show significant impacts on the bacterial diversity of the whole body of the parasitic wasp *N. vitripennis* (62). However, no reports focus on the gut microbe interaction between parasitoid wasp and its associated host. The tunneling and feeding activities of *P. xylostella* lead to the demise of the host, which could potentially result in the sharing of similar microbiota through transmission between parasitoids and *P. xylostella*. For example, based on beta diversity analysis, the gut microbiota of *C. vestalis* was found highly similar to that of *P. xylostella*. Moreover, *Enterococcus* was found to be a high abundance of members of the gut microbiome in these two parasitoid wasps, and it was also the dominant gut bacteria of the host. The presence of shared gut microbes between host and parasitoid wasps indicates the interaction of gut microbes. Despite the richness of microbial species and communities, no significant difference in the gut microbial communities was found between individuals of *C. vestalis* and *D. collaris*, and the gut microbial species of the parasitoids showed great overlap. This phenomenon was probably due to the two parasitoid species attacking different developmental stages of the same host species. Considering their potential functions, the shard gut microbes, especially the predominate bacteria, between host and parasitoid wasps may act as potential catalysts for the co-evolution of the host-parasite system.

In conclusion, the results revealed a detailed investigation of the gut microbial composition, diversity, and functional roles associated with the parasitoids *C. vestalis* and *D. collaris* that are associated with a common host. Our results showed a higher microbial richness in *C. vestalis* that parasitizes the larval host, while more diverse communities with greater richness and abundance in *D. collaris* parasitize the pupal host. The microbial species showed great overlap, and the gut microbial composition was similar between the two parasitoid wasps, suggesting diet is an important determinant of gut microorganisms. We found the predominant bacteria *Enterococcus* in the parasitoids and their host was similar, suggesting a possible link of gut microbes between parasitoid and their associated hosts. Functional analysis indicated the microbial functions associated with parasitoids may be related to the nutrient compositions of their diets. Our study characterized the gut microbiota of the parasitoids, its role in wasp nutrition, and the correlation with host gut microbiota, potentially paving the way for the development of an ecologically friendly biocontrol strategy against the pest *P. xylostella*.

## Data Availability

Raw sequence data are available on NCBI Sequence Read Archive (BioProject accession no. PRJNA1067415 and PRJNA1067380).

## SUPPLEMENTAL MATERIAL

Supplemental material spectrum.01208-24-s0001.docx Tables S1 to S8.

ASM does not own the copyrights to Supplemental Material that may be linked to, or accessed through, an article. The authors have granted ASM a non-exclusive, world-wide license to publish the Supplemental Material files. Please contact the corresponding author directly for reuse.
