# Supplementary material for: Characterization of larval gut microbiota of two endoparasitoid wasps associated with their common host, Plutella xylostella (Linnaeus) (Lepidoptera: Plutellidae)
Source: Microbiol Spectr. 2024 Sep 9;12(10):e01208-24. doi: 10.1128/spectrum.01208-24 (PMC11448028; doi:10.1128/spectrum.01208-24)
Supplement: Supplemental material — Tables S1 to S8. [file spectrum.01208-24-s0001.docx]

**Supplementary Tables**

**Supplementary Table S1** Summary of shotgun sequencing data from the whole gut metagenome of larval *C. vestalis* (Cv) and *D. collaris* (Dc).

| Samples | Sequence size  (Gbp) | Number of reads | Number of ORFs | Means length of ORFs (bp) |
| --- | --- | --- | --- | --- |
| Cv-1 | 27.512 | 183,414,212 | 28,595 | 402.32 |
| Cv-2 | 23.891 | 159,278,416 | 29,838 | 411.87 |
| Cv-3 | 27.383 | 182,554,634 | 28,590 | 412.99 |
| Dc-1 | 14.285 | 95,232,338 | 128,680 | 276.16 |
| Dc-2 | 14.296 | 95,302,466 | 128,131 | 272.47 |
| Dc-3 | 14.582 | 97,212,754 | 134,121 | 281.38 |

| **Supplementary Table S2** The numbers of microbial species from the larval gut of *C. vestalis* (Cv) and *D. collaris* (Dc) at different classification levels. | | |
| --- | --- | --- |
| Taxon | Cv | Dc |
| Phylum | 22 | 23 |
| Class | 39 | 49 |
| Order | 81 | 100 |
| Family | 152 | 163 |
| Genus | 330 | 298 |
| Species | 720 | 590 |

**Supplementary Table S3** Alpha diversity measures for the gut microbiota of the larval *C. vestalis* (Cv) and *D. collaris* (Dc) ( two-tailed unpaired *t* test)*.*

| Index | Degree of freedom | *R*-squared | Difference between means (Dc-Cv)  ± SEM | 95% confidence interval | P value |  |
| --- | --- | --- | --- | --- | --- | --- |
|  |  |  |  |  |  |  |
| Chao1 | 4 | 0.8734 | -199.0 ± 37.88 | -304.2 to -93.85 | 0.0063 |  |
| ACE | 4 | 0.9589 | -293.8 ± 30.41 | -378.3 to -209.4 | 0.0006 |  |
| Shannon | 4 | 0.3349 | 0.2368 ± 0.1669 | -0.2265 to 0.7001 | 0.2288 |  |
| Simpson | 4 | 0.8168 | 0.0331 ± 0.0078 | 0.0114 to 0.0549 | 0.0134 |  |

**Supplementary Table S4** PERMANOVA analyses on the gut microbial communities for the larval *C. vestalis* (Cv) and *D. collaris* (Dc) at different classification levels.

| Taxon | Degree of freedom | Sum of squares | *F* statistics | *R*-squared | P value |
| --- | --- | --- | --- | --- | --- |
| Phylum | 1 | 0.116 | 38.00 | 0.905 | 0.1 |
| Class | 1 | 0.168 | 14.35 | 0.782 | 0.1 |
| Order | 1 | 0.521 | 36.94 | 0.902 | 0.1 |
| Family | 1 | 0.593 | 40.87 | 0.911 | 0.1 |
| Genus | 1 | 0.610 | 40.63 | 0.910 | 0.1 |
| Species | 1 | 0.642 | 39.80 | 0.909 | 0.1 |

| **Supplementary Table S5** The numbers of microbial species shared and unique between the larval gut of *C. vestalis* (Cv) and *D. collaris* (Dc) at different classification levels. | | | |
| --- | --- | --- | --- |
| Taxon | Shared microbes | Unique microbes | |
|  |  | Cv | Dc |
| Phylum | 18 | 4 | 5 |
| Class | 28 | 11 | 21 |
| Order | 55 | 26 | 45 |
| Family | 90 | 62 | 73 |
| Genus | 159 | 171 | 139 |
| Species | 277 | 443 | 313 |

**Supplementary Table S6** The five dominant microbial species shared and unique between *C. vestalis* (Cv) and *D. collaris* (Dc) at different classification levels.

| Shared microbes | Unique microbes | |
| --- | --- | --- |
|  | Cv | Dc |
| Phylum |  |  |
| Proteobacteria  (43.685^a^, 52.386^b^) | Synergistetes  (<0.01) | Tenericutes  (0.057) |
| Firmicutes  (7.958^a^, 20.69^b^) | Deferribacteres  (<0.01) | Mucoromycota  (0.045) |
| Ascomycota  (0.153^a^, 2.78^b^) | Gemmatimonadetes  (<0.01) | Unclassified_Dikarya  (0.036) |
| Actinobacteria  (0.392^a^, 0.556^b^) | Spirochaetes  (<0.01) | Deinococcus-Thermus (0.019) |
| Apicomplexa  (0.354^a^, 0.494^b^) |  | Chlorobi  (0.017) |
| Class |  |  |
| Alphaproteobacteria  (26.544^a^, 35.006^b^) | Sphingobacteriia  (0.018) | Leotiomycetes  (0.192) |
| Gammaproteobacteria  (15.823^a^, 14.354^b^) | Eustigmatophyceae  (0.014) | Oomycetes  (0.137) |
| Bacilli  (7.92^a^, 20.527^b^) | Epsilonproteobacteria  (0.01) | Agaricomycetes  (0.109) |
| Betaproteobacteria  (0.611^a^, 2.003b) | Synergistia  (0.009) | Malasseziomycetes (0.088) |
| Eurotiomycetes  (0.006^a^, 1.881^b^) | Bacteroidia  (0.007) | Mollicutes  (0.057) |
| Order |  |  |
| Lactobacillales  (7.847^a^, 20.385^b^) | Vibrionales  (10.336) | Eurotiales  (1.595) |
| Rhizobiales  (19.365^a^, 4.066^b^) | Rickettsiales  (0.078) | Oscillatoriales  (0.346) |
| Sphingomonadales  (1.514^a^, 17.321^b^) | Cellvibrionales (0.033) | Ortervirales (0.185) |
| Enterobacterales  (1.435^a^, 13.266^b^) | Pasteurellales (0.029) | Caudovirales (0.158) |
| Rhodospirillales  (0.132^a^, 10.776^b^) | Methylococcales (0.024) | Hypocreales (0.112) |
| Family |  |  |
| Enterococcaceae  (7.847^a^, 20.236^b^) | Vibrionaceae  (10.336) | Aspergillaceae  (1.554) |
| Sphingomonadaceae  (1.489^a^, 17.217^b^) | Moraxellaceae  (0.54) | Saccharospirillaceae  (0.369) |
| Rhizobiaceae  (17.78^a^, 0.216^b^) | Shewanellaceae  (0.321) | Oscillatoriaceae  (0.346) |
| Yersiniaceae  (0.234^a^, 12.875^b^) | Erwiniaceae  (0.098) | Metaviridae  (0.185) |
| Acetobacteraceae  (0.024^a^, 10.492^b^) | Anaplasmataceae  (0.078) | Siphoviridae  (0.158) |
| Genus |  |  |
| *Enterococcus*  (7.847^a^, 20.236^b^) | *Photobacterium*  (6.225) | *Asaia*  (8.884) |
| *Rhizobium*  (14.776^a^, 0.052^b^) | *Vibrio* (3.204) | *Aspergillus* (1.517) |
| *Serratia*  (0.167^a^, 12.871^b^) | *Acinetobacter*  (0.538) | *Komagataeibacter*  (0.738) |
| *Sphingobium*  (0.065^a^, 10.241^b^) | *Shewanella*  (0.321) | *Gynuella*  (0.369) |
| *Caulobacter*  (3.366^a^, 1.939^b^) | *Grimontia*  (0.147) | *Moorea*  (0.331) |
| Species |  |  |
| *Enterococcus rotai*  (5.009^a^, 15.387^b^) | *Photobacterium gaetbulicola* (3.935) | *Asaia bogorensis*  (8.884) |
| *Sphingobium yanoikuyae* (0.024^a^, 8.627^b^) | *Photobacterium profundum* (1.366) | *Komagataeibacter europaeus* (0.632) |
| *Rhizobium phaseoli*  (5.581^a^, 0.007^b^) | *Aeromonas veronii*  (0.932) | *Acidovorax* sp. RAC01 (0.609) |
| *Serratia marcescens*  (0.052^a^, 4.959^b^) | *Acinetobacter johnsonii* (0.406) | *Sphingobium* sp. YG1 (0.593) |
| *Enterococcus faecalis*  (1.162^a^, 1.451^b^) | *Plasmodium berghei*  (0.308) | *Aspergillus oryzae*  (0.566) |

Number in bracket indicates the percentage of microbes.

a, and b indicate the percentage of the common microbes in *C. vestalis* and *D. collaris*, respectively.

| **Supplementary Table S7** Correlation patterns showing association between two predominant genera and all KEGG pathways within *C. vestalis* (Cv) group. | | | | |
| --- | --- | --- | --- | --- |
| KEGG pathway (Cv) | *Rhizobium* | | *Enterococcus* | |
|  | R value | P value | R value | P value |
| Carbohydrate metabolism | -0.5 | 0.667 | 1 | 0.000 |
| Amino acid metabolism | -0.5 | 0.667 | 1 | 0.000 |
| Membrane transport | -0.5 | 0.667 | 1 | 0.000 |
| Energy metabolism | -0.5 | 0.667 | 1 | 0.000 |
| Nucleotide metabolism | -0.5 | 0.667 | 1 | 0.000 |
| Signal transduction | 0.5 | 0.667 | -1 | 0.000 |
| Metabolism of cofactors and vitamins | 0.5 | 0.667 | 0.5 | 0.667 |
| Cellular community - prokaryotes | -0.5 | 0.667 | 1 | 0.000 |
| Lipid metabolism | -0.5 | 0.667 | 1 | 0.000 |
| Translation | -0.5 | 0.667 | 1 | 0.000 |
| Replication and repair | -0.5 | 0.667 | 1 | 0.000 |
| Xenobiotics biodegradation and metabolism | 1 | 0.000 | -0.5 | 0.667 |
| Folding, sorting and degradation | -0.5 | 0.667 | 1 | 0.000 |
| Glycan biosynthesis and metabolism | 0.5 | 0.667 | -1 | 0.000 |
| Endocrine system | 0.5 | 0.667 | -1 | 0.000 |
| Drug resistance: Antimicrobial | -0.5 | 0.667 | 1 | 0.000 |
| Metabolism of terpenoids and polyketides | -0.5 | 0.667 | 1 | 0.000 |
| Cancers: Overview | 0.5 | 0.667 | -1 | 0.000 |
| Neurodegenerative diseases | 0.5 | 0.667 | -1 | 0.000 |
| Cell growth and death | 1 | 0.000 | -0.5 | 0.667 |
| Digestive system | 0.5 | 0.667 | -1 | 0.000 |
| Infectious diseases: Parasitic | 0.5 | 0.667 | -1 | 0.000 |
| Transport and catabolism | 0.5 | 0.667 | -1 | 0.000 |
| Infectious diseases: Bacterial | 1 | 0.000 | -0.5 | 0.667 |
| Cardiovascular diseases | 0.5 | 0.667 | -1 | 0.000 |
| Aging | 0.5 | 0.667 | -1 | 0.000 |
| Cell motility | 1 | 0.000 | -0.5 | 0.667 |
| Immune system | 0.5 | 0.667 | -1 | 0.000 |
| Infectious diseases: Viral | 0.5 | 0.667 | -1 | 0.000 |
| Cancers: Specific types | 0.5 | 0.667 | -1 | 0.000 |
| Nervous system | 0.5 | 0.667 | -1 | 0.000 |
| Endocrine and metabolic diseases | 0.5 | 0.667 | -1 | 0.000 |
| Drug resistance: Antineoplastic | 0.5 | 0.667 | -1 | 0.000 |
| Environmental adaptation | 0.5 | 0.667 | -1 | 0.000 |
| Transcription | 0.5 | 0.667 | -1 | 0.000 |
| Development | 0.5 | 0.667 | -1 | 0.000 |
| Cellular community - eukaryotes | 0.5 | 0.667 | -1 | 0.000 |
| Immune diseases | -0.5 | 0.667 | 1 | 0.000 |
| Substance dependence | 0.5 | 0.667 | -1 | 0.000 |
| Signaling molecules and interaction | 0.5 | 0.667 | -1 | 0.000 |
| Sensory system | 0.5 | 0.667 | -1 | 0.000 |
| Excretory system | 0.5 | 0.667 | -1 | 0.000 |
| Circulatory system | 0.5 | 0.667 | -1 | 0.000 |

| **Supplementary Table S8** Correlation patterns showing association between two predominant genera and all KEGG pathways within *D. collaris* (Dc) group. | | | | |  |
| --- | --- | --- | --- | --- | --- |
| KEGG pathway (Dc) | *Enterococcus* | | *Serratia* | |  |
|  | R value | p value | R value | p value |  |
| Carbohydrate metabolism | 0.5 | 0.667 | -1 | 0.000 |  |
| Signal transduction | -0.5 | 0.667 | 1 | 0.000 |  |
| Amino acid metabolism | 0.5 | 0.667 | -1 | 0.000 |  |
| Endocrine system | -0.5 | 0.667 | 1 | 0.000 |  |
| Translation | -0.5 | 0.667 | 1 | 0.000 |  |
| Membrane transport | 0.5 | 0.667 | -1 | 0.000 |  |
| Nucleotide metabolism | 0.5 | 0.667 | -1 | 0.000 |  |
| Cancers: Overview | -0.5 | 0.667 | 1 | 0.000 |  |
| Cell growth and death | -0.5 | 0.667 | 1 | 0.000 |  |
| Lipid metabolism | -0.5 | 0.667 | 1 | 0.000 |  |
| Infectious diseases: Viral | -0.5 | 0.667 | 1 | 0.000 |  |
| Metabolism of cofactors and vitamins | 0.5 | 0.667 | -1 | 0.000 |  |
| Transport and catabolism | -0.5 | 0.667 | 1 | 0.000 |  |
| Energy metabolism | 0.5 | 0.667 | -1 | 0.000 |  |
| Cancers: Specific types | -1 | 0.000 | 0.5 | 0.667 |  |
| Replication and repair | 1 | 0.000 | -0.5 | 0.667 |  |
| Folding, sorting and degradation | -0.5 | 0.667 | 1 | 0.000 |  |
| Neurodegenerative diseases | -0.5 | 0.667 | 1 | 0.000 |  |
| Xenobiotics biodegradation and metabolism | 0.5 | 0.667 | -1 | 0.000 |  |
| Immune system | -0.5 | 0.667 | 1 | 0.000 |  |
| Endocrine and metabolic diseases | -0.5 | 0.667 | 1 | 0.000 |  |
| Cellular community - prokaryotes | 0.5 | 0.667 | -1 | 0.000 |  |
| Glycan biosynthesis and metabolism | 0.5 | 0.667 | -1 | 0.000 |  |
| Digestive system | -0.5 | 0.667 | 1 | 0.000 |  |
| Infectious diseases: Bacterial | -0.5 | 0.667 | 1 | 0.000 |  |
| Cellular community - eukaryotes | -0.5 | 0.667 | 1 | 0.000 |  |
| Nervous system | -0.5 | 0.667 | 1 | 0.000 |  |
| Aging | -1 | 0.000 | 0.5 | 0.667 |  |
| Transcription | -0.5 | 0.667 | 1 | 0.000 |  |
| Environmental adaptation | -0.5 | 0.667 | 1 | 0.000 |  |
| Cardiovascular diseases | -0.5 | 0.667 | 1 | 0.000 |  |
| Metabolism of terpenoids and polyketides | 0.5 | 0.667 | -1 | 0.000 |  |
| Drug resistance: Antineoplastic | -0.5 | 0.667 | 1 | 0.000 |  |
| Infectious diseases: Parasitic | -0.5 | 0.667 | 1 | 0.000 |  |
| Development | -0.5 | 0.667 | -0.5 | 0.667 |  |
| Drug resistance: Antimicrobial | 0.5 | 0.667 | -1 | 0.000 |  |
| Signaling molecules and interaction | -0.5 | 0.667 | 1 | 0.000 |  |
| Excretory system | -0.5 | 0.667 | 1 | 0.000 |  |
| Circulatory system | -0.5 | 0.667 | 1 | 0.000 |  |
| Substance dependence | -0.5 | 0.667 | 1 | 0.000 |  |
| Cell motility | -0.5 | 0.667 | -0.5 | 0.667 |  |
| Sensory system | -0.5 | 0.667 | 1 | 0.000 |  |
| Immune diseases | 1 | 0.000 | -0.5 | 0.667 |  |
